# Comparison Study on the Trophic Niche of Red Pandas Using Stable Isotope Analysis

**DOI:** 10.3390/ani14233512

**Published:** 2024-12-05

**Authors:** Yuyu Zhang, Wanxin Lei, Wei Luo, Qinlong Dai, Han Han, Yonggang Nie

**Affiliations:** 1 Key Laboratory of Southwest China Wildlife Resources Conservation (Ministry of Education), China West Normal University, Nanchong 637009, China; zyy18209444413@163.com (Y.Z.); lwx1183606568@163.com (W.L.); 2 Giant Panda College, China West Normal University, Nanchong 637009, China; 3 Liziping Giant Panda’s Ecology and Conservation Observation and Research Station of Sichuan Province, Nanchong 637009, China; pandaluowei@aliyun.com (W.L.); qinlongdai@163.com (Q.D.); 4 Institute of Zoology, Chinese Academy of Sciences, Beijing 100101, China

**Keywords:** red pandas, stable isotopes, trophic niche, Bayesian ellipse, niche width

## Abstract

The red panda (Ailurus), a rare and endangered mammal native to the Himalayan–Hengduan Mountains, has a specialized bamboo diet. Combining morphological and genomic evidence, red pandas have been classified as *Ailurus fulgens* and *Ailurus styani*. However, previous studies focused on ecological aspects such as foraging behaviors, habitat use and threats within specific distributions; hence, there is still a gap in quantitative comparative studies on the trophic niches of these two species. In this study, we calculated and compared the isotopic trophic niche widths of *A. styani* and *A. fulgens* by measuring carbon and nitrogen stable isotopes in hair keratin of 102 red pandas, then conducting a fecal analysis to explore the diet of a population. The results showed that (1) there was no significant difference in δ^13^C values between *A*. *styani* (−24.73 ± 1.26‰, N = 86) and *A. fulgens* (−24.43 ± 1.20‰, N = 16); however, *A*. *styani* had 1.4‰ higher average δ^1^⁵N value than that of *A. fulgens,* and the isotopic niche widths converted by Bayesian ellipse model were *A*. *styani* SEA_C_ = 4.40‰^2^, *A. fulgens* SEA_C_ = 4.70‰^2^, which might be caused by their lower and narrower altitude distribution. (2) Niche widths of three genetic populations in *A*. *styani* were XXL-LS (5.16‰^2^), EH-GLG (3.40‰^2^), QL (2.31‰^2^), and the comparison of the fecal composition suggested their diet diversity, which might imply a degree of competitive pressure with sympatric animals. (3) Sichuan red pandas occupied a lower trophic position compared to other herbivores but higher than giant pandas in the same ecosystem, and the niche width of red pandas was two times larger than that of sympatric giant pandas, which was related to the foraging strategies and microhabitat selection of the giant and red panda. This research provides basic stable isotopic data for red pandas, offers scientific support for the differentiated and refined conservation and management of the red panda and its habitat, and develops the application of stable isotope analysis in the ecological studies of endangered species.

## Simple Summary

The red panda, with an exclusive bamboo diet, has been classified as *Ailurus fulgens* and *Ailurus styani*. However, the quantitative comparison of the trophic niches of these two species is still an unsolved problem. Using stable isotope analysis, we found that *Ailurus styani* had similar δ^13^C values in the hair keratin to *Ailurus fulgens*, but significantly higher δ^15^N values due to different altitude distribution. Furthermore, the two red panda species had comparable trophic niche widths. Three genetic populations of *A. styani* endured various levels of competitive pressure. The Sichuan red panda occupied a relative lower trophic level than local carnivores and herbivores, but a bit higher than giant pandas, and the foraging strategies and habitat partition was attributed to the difference of niche width of the two pandas. We suggest more differentiated and refined conservation and management of the red panda and its habitat, and this study provides a new insight into intraspecific trophic niche studies.

## 1. Introduction

The red panda is a small arboreal mammal with a carnivorous taxonomy, but over the course of evolution, it has become a strict herbivore, feeding mainly on bamboo [1]. However, the red panda still retains the gastrointestinal system characteristics of a typical carnivore [2], which limits its ability to meet nutritional needs through cellulose digestion. Being a high-cellulose and low-protein food resource, bamboo modified the red panda, leading to a unique lifestyle for survival and reproduction [3]. The specialization of the red panda’s diet and the development of adaptive features in its morphology, physiology, behavior, and ecology make it a suitable subject for scientific research [4]. Red pandas primarily live in the Himalayan–Hengduan Mountains and their neighboring regions, including bamboo forests in extensive temperate and coniferous forest areas, and adjacent broadleaf forests from Nepal along the Himalayas eastward through northern Myanmar to southwestern China [5]. Despite having a diet and distribution similar to that of the giant panda, the red panda has received considerably less attention and research effort [6]. For example, after a painting from Europe introduced in 1821 the mysterious red panda [7] to the world [8,9], research on the red panda stagnated until the 1970s, when China conducted the “First National Survey of the Giant Panda” program [10]. Previous studies conducted on wild red pandas mainly focused on their biological characteristics (e.g., foraging strategies, life rhythms, and reproductive behaviors), population dynamics and genetic diversity, habitat selection and use, and threatening factors (e.g., habitat loss or fragmentation, poaching, illegal trade, and human activities) [11,12,13,14,15,16,17,18,19,20]. Most of them compared red pandas with giant pandas or other sympatric animals, and few targeted studies on different red panda populations in various mountains. The distribution range of wild red pandas has shrunk significantly in recent years, impacting their long-term survival. Consequently, they are listed as “Endangered” by the IUCN [21,22]. Anthropogenic disturbances have had a severe impact on red panda habitat, including deforestation, infrastructure development and grazing, which has led to habitat fragmentation, uneven distribution of food resources, and reduced adaptability to the environment, making the species more vulnerable [23], ultimately increasing the extinction risk of local populations [24,25]. The lack of knowledge of the status of different red panda populations hinders the development of long-term and refined conservation management policies.

The niche is a core concept and plays an important role in ecology. Traditional study methods of ecological niche usually concerned specific resource scales. Each method emphasizes one particular aspect of the species’ ecological characteristics, which neither comprehensively represents the multidimensional ecological niche nor allows for interspecific or infraspecific population comparisons. As a relatively new tool used in ecology, stable isotope analysis (SIA) has many advantages such as tracing, integration and indication in the environment, as well as rapid detection and accurate results. Therefore, the SIA method has been widely applied by ecologists for niche measurement, inter- and intraspecific dietary diversity [26], resource contribution [27], species invasions [28], and niche variation along temporal and spatial gradients [29,30]. In particular, the bivariate isotopic ratio plot, which is commonly composed of δ^13^C and δ^1^⁵N, can indicate multidimensional eco-spatial information including both biotic resources and abiotic habitats. They can be used to quantify and compare trophic niche differences within or among species [31,32,33]. Researchers combined stable isotope (SI) and fatty acid (FA) analysis of muscle tissue with morphometric measurements of feeding organs to assess spatial patterns of habitat and resource use by *Dosidicus gigas* collected from three major fisheries in the tropical and southern temperate Pacific Ocean [34]. Additionally, trophic levels of animals can be determined through individual carbon and nitrogen isotope analysis of amino acids [35]. Furthermore, the SIA method is extremely suitable for studying wild populations of rare and endangered species due to its non-invasive sampling and the small amounts required for measurement [36].

The stable isotopic studies of giant pandas have found that two different genetic populations occupied distinct trophic positions in their respective ecosystems, but their niche widths differed significantly [37,38]. Red pandas, which have highly similar diets and distribution to those of giant pandas, are classified into two species—*Ailurus fulgens* (Himalayan red panda) and *Ailurus styani* (Chinese red panda), separated by the Yarlung Zangbo River [39]. Additionally, *A*. *styani* can be divided into three genetic populations: Eastern Himalaya-Gaoligong (EH-GLG), Xiaoxiangling-Liangshan (XXL-LS) and Qionglai (QL) [1]. In this study, we determined the carbon and nitrogen stable isotope ratios of red pandas’ hair keratin and accurately quantified and compared the trophic niche width and overlap of two red pandas (*A. styani* and *A. fulgens*) with a Bayesian mixing model, as well within Chinese red pandas. Food sources, whether regular or seasonal, are crucial to the survival of animals, so we conducted fecal analysis of red pandas to discuss the diet, which may be the major factor affecting the trophic niche width of different red panda populations. Then, we confirmed the trophic position of Chinese red pandas in their habitat, and explored the underlying mechanisms of niche formation. Furthermore, we compared the trophic positions and stable isotopic niche widths of giant pandas and red pandas, investigating the competition and coexistence mechanisms between these two species. This study provides suggestions for the protection and management of red pandas.

## 2. Materials and Methods

### 2.1. Sample Collection

**Figure 1:**
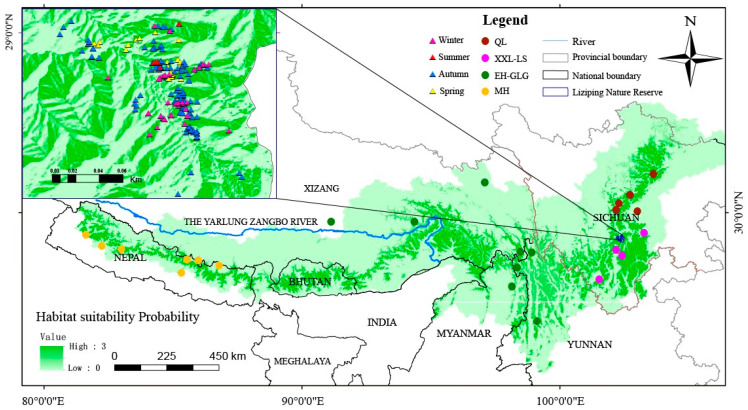
The geographic distribution of red panda hair samples (dots) and fecal collections (triangles) under the background of habitat suitability probability. The potential suitable habitat areas were redrawn from the published paper [1].

The chemical composition of food is integrated into different tissues when it enters into the animal’s body. The stable isotopic composition of animal tissues is related to the food they consumed and each tissue represents the animal’s diet over different time scales (from a few hours to a whole life) in terms of their different metabolic rates. Hairs can reflect the stable isotopic composition of the individual’s diet over compatible time periods, and their collection does not cause damage to the live animal or specimens. We obtained a large number of hair samples of red pandas from fur specimens reserved in libraries and occasionally picked up some from places where red pandas foraged and slept in the field when patrolling (Figure 1). Samples of Himalayan red pandas were mainly from Nepal, while Chinese red pandas were divided into three populations: EH-GLG, XXL-LS and QL, according to their genetic background. Coarse hairs were selected and taken from the same location (excluding hair follicles) and brought back to the laboratory in centrifuge tubes for pretreatment. We collected one of the dropping groups (multiple defecation) or a single dropping of red pandas for fecal analysis opportunistically encountered along the survey trails in different places in the wild over the period of a year from Liziping National Nature Reserve in Xiaoxiangling Mountain (Figure 1, enlarged part). The feces were oven-dried, and components such as stems, leaves, shoots, and fruits were identified and weighed. The food items in the droppings were not completely digested and thus could be easily separated. The proportion of food composition in the diet was calculated based on the weight of dry matter.

### 2.2. Laboratory Methods

The pre-treatment process for hair samples included washing, degreasing and drying. The samples were rinsed with deionized water at first to remove impurities such as dirt and dust. The hair contains a small amount of lipids, and they can greatly affect the δ^13^C values [38,40]. We used acetone to degrease, eliminating the negative effect of lipids. Then, the hair samples were rinsed with ultrapure water again to remove excess acetone, and finally placed in an oven at 50 °C to get rid of water. The fecal samples were brought back to the laboratory and dried in batches in an oven at 60 °C until constant weight, and sieved through a 60-mesh sieve so that we could distinguish all the constituents.

### 2.3. Stable Isotope Analysis

Carbon and nitrogen stable isotope ratios of samples were determined in the Stable Isotope Analysis Laboratory at the Institute of Zoology, Chinese Academy of Sciences. Hair samples were cut into small segments and mixed well. About 0.2~0.5 mg of evenly distributed mixture was weighed by one-millionth microbalance (XP6 electronic balance, Mettler-Toledo, Zurich, Switzerland) and then wrapped in a tin boat and dried for testing. The samples were purified and separated by the elemental analyzer using the combustion method; the carbonates in the samples were cracked into carbon dioxide at high temperatures, while the nitrogen was converted into gas nitrogen, and the purified and dried gases were transferred to a stable isotope ratio mass spectrometer (IRMS, Thermo Scientific 253 Plus, Thermo fisher Scientific, Waltham, MA, USA) for isotopic value determination. N_2_ and CO_2_ were used as reference gases, respectively, for carbon and nitrogen, and calibrated by multipoint segmented calibration curves of reference materials B2157 and B2159 (wheat and sorghum flour, Elemental Microanalysis, Okehampton, Devon, UK). Laboratory standards were inserted every 12 samples during the analysis for quality control. The laboratory standard was D-Phenylalanine (Sigma-Aldrich, St. Louis, MI, USA) with a long-term standard deviation of 0.2‰ for both carbon and nitrogen.

### 2.4. Statistical Analysis

All data were analyzed using descriptive statistics (means) and ANOVA models for one-way analysis of variance (ANOVA) as well as multiple comparisons using the describe function in the psych package of the R language (R4.3.2) software [41]. Significant differences were set at *p* ≤ 0.05. We corrected the carbon isotope values of hair keratin for the Suess effect and used the corrected values in subsequent statistical analyses [42,43]. Stable isotope trophic niche width was converted using the Stable Isotope Bayesian Ellipses (SIBER) package in R software to calculate niche widths and overlap [44]. Results of stable isotope ratios are presented as mean and standard deviation (mean ± SD).

## 3. Results

### 3.1. Characterization of Two Red Panda Species

**Figure 2:**
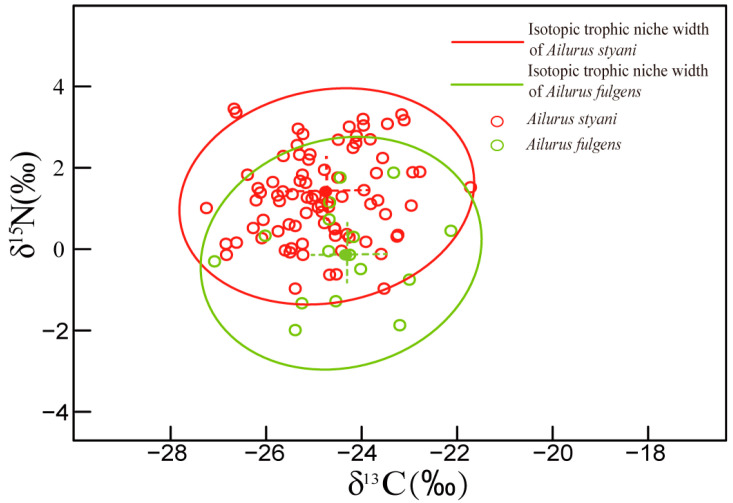
Carbon and nitrogen stable isotope ratios and trophic niche ellipses of *Ailurus styani* (red circles) and *Ailurus fulgens* (green circles).

Carbon and nitrogen stable isotope data from a total of 102 red panda hair samples were determined, including 86 *A*. *fulgens* and 16 *A. styani* (Table 1). There were no significant differences in carbon isotopes between Himalayan and Chinese red panda populations (F (1) = 0.772, *p* > 0.05); however, *A. styani* was 1.4‰ more enriched in δ^15^N than *A. fulgens* (F (1) = 21.77, *p* < 0.001) (Figure 2). We converted the carbon and nitrogen stable isotope variance of these two red pandas into ellipses by a Bayesian mixing model SIBER. The ellipse represented the trophic ecological niches, and the widths of their trophic niches were quantified by calculating areas of the ellipses. The results showed that the area of the Bayesian ellipse composed of δ^13^C and δ^15^N from *A*. *styani* was SEA_B_ = 4.27‰^2^, and a corrected value for small sample size SEA_C_ = 4.40‰^2^, while the stable isotopic trophic niche width of *A*. *fulgens* was SEA_B_ = 4.38‰^2^, and SEA_C_ = 4.70‰^2^ after correction. The difference of the niche widths between the two red panda species did not reach the significance level (*p* = 0.56). The overlapping area of these two isotopic ellipses occupied 70% of the trophic niche width of *A*. *styani* and 65% of that of *A. fulgens* (Figure 2).

### 3.2. Different Populations Within the Red Panda Species

**Figure 3:**
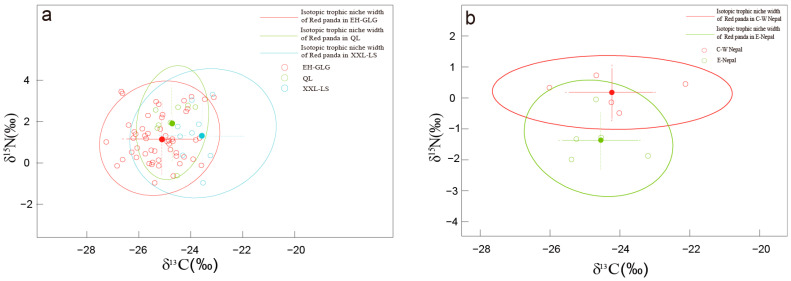
Carbon and nitrogen isotopic niche ellipses within two red panda species. (**a**). *Ailurus styani* (red: EH-GLG, blue: XXL-LS, and green: QL populations); (**b**). *Ailurus fulgens* (red: C-W Nepal and green: E-Nepal populations).

In order to explore the stable isotopic variation within the species of red pandas, hair samples from *A. styani* were divided into three groups: EH-GLG (including samples from Myanmar, Yunnan and Xizang Province in China), XXL-LS (Xiaoxiangling and Liangshan Mountain), and QL (Qionglai Mountain) (Table 1). Statistical analyses showed that there were no significant differences in δ^15^N values among the three populations (*p* > 0.05). However, for carbon stable isotopes, the difference between the EH-GLG and XXL-LS populations reached a highly significant level (*p* < 0.01); at the same time, the δ^13^C values of the hairs from the XXL-LS was different from the QL population (*p* < 0.05), while the disparity of GLG and QL populations was not evident (*p* > 0.05) in carbon isotopes (Figure 3a). The trophic niche ellipses of three Chinese red panda genetic populations were constructed based on the carbon and nitrogen isotopes of their hair keratin through the SIBER model, and their niche widths were indicated using areas of the ellipses. The model carried out 10^6^ sampling iterations on the basis of the Bayesian method, respectively, to obtain the stable isotopic trophic niche width, SEA_B_, and correction for small sample sizes, SEA_C_, for EH-GLG, XXL-LS and QL red pandas. The results demonstrated that the isotopic trophic niche width of the XXL-LS population was largest (SEA_B_ = 4.59‰^2^, SEA_C_ = 5.16‰^2^), followed by the EH-GLG group: SEA_B_ = 3.33‰^2^, SEA_C_ = 3.40‰^2^, then QL red pandas have the smallest trophic niche ellipse area: SEA_B_ = 1.99‰^2^ (SEA_C_ = 2.31‰^2^). There is overlap in the areas among three red panda populations. The overlap area between the XXL-LS and EH-GLG occupied 75% of EH-GLG and 49% of XXL-LS; 42% of QL and 93% of XXL-LS areas made up their overlap; the overlapping area between EH-GLG and QL, respectively, took up 59% and 86% of EH-GLG and QL ellipses.

The conservation and management of Chinese red pandas, especially the populations in Sichuan Province, have always relied on the strong network established for the giant panda. Therefore, the populations are protected by mountains, following the distribution of giant pandas. In light of this, we tried to split the XXL-LS genetic population of *A*. *styani* into two groups: XXL Mountain (XXLM) and LS Mountain (LSM). The carbon stable isotope ratios of hair keratin from the XXLM red panda fluctuated between −24.41‰ and −23.16‰, with a mean value of −23.84 ± 0.53‰ (N = 6), and the δ^15^N values varied from 0.35‰ to 3.31‰, and the average value was 1.63 ± 1.28‰ (N = 6). And δ^13^C values of the LSM population ranged from −24.50‰ to −20.53‰ (mean: −23.06 ± 1.74‰, N = 4), while the distribution of δ^15^N values was from −0.97‰ to 1.87‰, with a mean of 1.12 ± 1.40‰ (N = 4). With the addition of the QL genetic population, which is also located in Sichuan Province, the statistical analysis revealed that there was no significant difference in nitrogen isotopes among these three Sichuan red pandas (df = 2, *p* > 0.05), whereas significant differences were found for δ^13^C values (QL vs. XXLM: t = −2.894, df = 12, *p* < 0.05; QL vs. LSM: U = 29, *p* < 0.05), but the difference between XXLM and LLSM red pandas did not reach a significant level (t = −1.05, df = 8, *p* > 0.05). We collected 270 feces from Liziping National Nature Reserve to represent the XXLM population and analyzed their diet composition. The results showed that XXLM red pandas ate exclusively bamboo leaves in spring and winter, ingested nearly half of the bamboo shoots in summer, and consumed some berries and mosses in summer and fall (Figure S1).

Himalayan red pandas were divided into two populations, eastern (E-Nepal) and central-western (C-W Nepal), to explore the trophic niche situation of red pandas within Nepal. The carbon and nitrogen stable isotope ratios of *A*. *fulgens* hair keratin in E-Nepal and C-W Nepal are displayed in Table 1. There was no significant difference in carbon isotopes between E-Nepal and C-W Nepal red panda populations (F (1) = 0.28, *p* > 0.05); however, for δ^15^N, red pandas from C-W Nepal were higher than E-Nepal by 1.48‰ (F (1) = 13.15, *p* < 0.001). The trophic niche ellipses constructed by hair keratin carbon and nitrogen isotopes of these two Himalayan red panda populations were calculated as E-Nepal SEA_B_ = 2.08‰^2^ (SEA_C_ = 2.77‰^2^), and the C-W Nepal population was SEAB = 2.14‰^2^ and corrected SEA_C_ = 2.86‰^2^ (Figure 3b). The stable isotope trophic niche widths of these two red panda populations were not significantly different (*p* = 0.48).

### 3.3. Giant Pandas and Red Pandas

Giant pandas and red pandas share habitats in Sichuan (SC) Province. Therefore, we compared the stable isotope composition of red pandas (N = 18) measured in this paper with that of giant pandas (some data from [38], N = 24) from Sichuan. The carbon isotopic values of SC red pandas fluctuated from −25.35‰ to −20.53‰ (mean ± SD: −24.05 ± 1.09‰), while the δ^13^C values of giant pandas varied from −25.31‰ to −20.91‰ with a mean of −23.44 ± 1.12‰. The nitrogen isotopes of the red panda fluctuated between −0.97‰ and 3.31‰ with a median of 1.66 ± 1.21‰, while the δ^15^N values of the giant panda varied from −0.55‰ to 1.91‰ (mean ± SD: 0.80 ± 0.68‰). Both δ^13^C and δ^15^N values of red pandas were significantly different from those of giant pandas (δ^13^C: U = 302.5, *p* < 0.05, δ^15^N: t = 2.927, df = 40, *p* < 0.05). The trophic niche width of the SC red panda population was 4.14‰^2^ (SEA_B_), and the correction for small sample size was 4.40‰^2^ (SEAc); the ellipse area of the SC giant pandas was SEA_B_ = 2.03‰^2^, while that corrected for small sample size SEAc = 2.13‰^2^. The width of the isotopic trophic niche occupied by the red panda was two times larger than that of the giant panda. Moreover, the giant pandas’ stable isotopic niche ellipse was nearly included in that of red pandas (Figure S2). The overlapping area accounted for 92% of the area of the giant panda niche ellipse, which is related to the highly similar diets of these two species.

## 4. Discussion

Animals incorporate stable isotope information into their tissues through the ingestion of food, so the chemical composition of these tissues directly reflects that of their diet [45,46]. Early studies about carbon stable isotopes in animal tissues and food found that the carbon isotopic values in animal tissues closely matched those of the food they consumed [47,48,49]. Additionally, the nitrogen stable isotopes in food, once assimilated into the animal, tend to be enriched in their tissues [50,51]. The carbon isotope values of *A. fulgens* and *A. styani* were comparable, while *A. styani* exhibited higher δ^15^N values. Therefore, the primary source of the differences in the stable isotope composition of the hair keratin of these two red panda species is likely derived from the stable isotope composition of their diet—bamboo. In terrestrial ecosystems, the stable isotope composition of plants is influenced by various environmental factors, including plant type, temperature, altitude, and humidity. It has been shown that as altitude increases, δ^15^N values in soil would decline, and consequently, the δ^15^N in plant tissues also decreases [52]. A study in giant pandas based on stable isotopes has discovered that δ^15^N values in bamboo showed a negative correlation with altitude [38]. Another study on the stable isotopic characteristics of needles and soils in Nepal pine forests along altitudinal gradients observed a similar pattern [53]. *A*. *fulgens* lives at altitudes ranging from 1500 to 4800 m [54], while *A. styani* is commonly found at altitudes between 1400 and 3400 m [55]. Red pandas forage for plants growing within habitats at different altitudes, leading to differences in the nitrogen isotope composition of keratin nitrogen in their hair tissues. Analyzing the statistical relationship between nitrogen isotope and the altitude at which the red panda hair samples were collected was not possible because most of the exact locations of the red panda hair samples were unclear. Based on the idea that “you are what you eat”, the primary source of the differences in the stable isotope composition of animal tissues mostly originates in their food. Therefore, we resorted to comparing nitrogen isotopic values of their diet as a proxy instead.

The width of the isotopic trophic niche indicates the breadth of isotopic composition in food resources utilized by the species, and the wider its trophic niche, the more various isotopes in food. The carbon and nitrogen stable isotopic Bayesian ellipse area of *A. fulgens*, which represents the breadth of its trophic niche, was slightly larger than that of *A. styani*, likely due to its wider altitudinal distribution. For *A*. *styani*, the three genetic populations were sorted in order: XXL-LS, EH-GLG, QL, according to their stable isotope trophic niche widths. The intraspecies comparison of isotopic trophic niche may imply the competitive pressure the species endured. The larger ellipse area demonstrated that animals needed a wider niche and had greater resource competition with sympatric fauna; in contrast, the smaller area indicated that red pandas living there did not have to explore more feeding habitat to avoid other animals and occupied a narrower trophic niche width. Therefore, the XXL-LS population which had the largest trophic niche width might face greater food competitive pressures from sympatric species.

Combined with the fecal analysis of red pandas in different seasons from Meigu Dafengding National Nature Reserve in Liangshan Mountain [56], LS red pandas fed on bamboo leaves for more than 99% of their diet in spring and winter, bamboo shoots accounted for about 15.8% of the fecal composition of red pandas in summer, and berry foraging in fall reached about 8.6%. The fecal composition of the QL population was similar in species but different in proportion, with the distribution of their foraging being bamboo leaves (70.5%), bamboo shoots (22.1%), berries (7.2%), hair (0.2%) [57]. In the study of giant pandas, which also feed mainly on bamboo, there have been relevant results expressing that there were significant differences in carbon stable isotopes between different tissue parts of bamboo, while the δ^15^N values did not reach the level of differences [40]. Therefore, we hypothesize that the differences between different genetic populations of *A. styani* are due to the combined effects of their living environment and foraging patterns. It follows that even for the same species, slightly different feeding behaviors due to their habitat or competitive pressure could result in trophic niche width changing. In addition, when considering the effect of ingested food, we should not only consider food types that can be distinguished from stable isotopes, but also the effect of possible differences in different components of the food item consumed by the animal, especially for the large portion.

**Figure 4:**
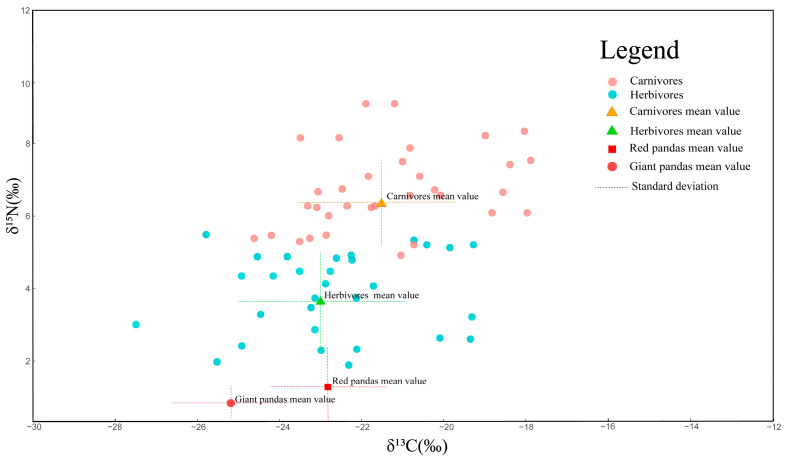
Carbon and nitrogen stable isotope distribution of red pandas and sympatric animals in Sichuan.

Carbon and nitrogen stable isotope values can determine an animal’s specific position in the food chain. As the trophic level rises, the carbon and nitrogen isotope composition of animal tissues change regularly. Studies have uncovered that, for each trophic level, δ^13^C is enriched by an average of 1–2‰ and δ^15^N is enriched by an average of 2–5‰, but nitrogen isotope fractionation is more stable [58,59]. We can determine the trophic position of an animal in the ecosystem by measuring carbon and nitrogen stable isotopes. However, there are some limits to using this method in terrestrial ecosystems, as the accurate determination of trophic levels requires a baseline (i.e., primary producers). It is hard to obtain the correct baseline for different regions, as plant resources vary. Nonetheless, herbivores will have lower δ^15^N values in their tissues than carnivores in the same ecosystem, and we can determine the relative trophic position of species. We compared the stable isotopic composition of hair from SC red pandas (including individuals from XXL-LS and QL) with that of other carnivores and herbivores, including giant pandas from the same distribution [60], to obtain the relative trophic position of the red panda in Sichuan Province. As can be visualized from the figure (Figure 4), the data exhibited a regular and clear increasing trend along the food chain, with carnivores having a higher relative trophic level than herbivores. However, as a species in Carnivora, the red panda was in a lower trophic position than any other carnivores and even herbivores. Coinciding with their highly overlapping diet, the red panda’s relative trophic position is close to that of the giant panda in the ecosystem, but slightly higher. Although both red and giant pandas have become exclusive bamboo eaters during the long evolutionary process, there are some differences, with red pandas foraging for a significant proportion of berries in the summer and fall, which have higher carbon and nitrogen stable isotope ratios than bamboo [36]. This may account for the slight difference in their relative trophic position.

The results of the isotopic analysis model showed that the trophic niche width of red pandas was larger than that of giant pandas, suggesting the more complicated food composition of red pandas. The large overlapping area of isotopic ellipses between giant pandas and red pandas in Sichuan indicated that they shared the same food, thus indicating a certain competitive pressure: both special carnivores exclusively feeding on bamboo and occupying the same regions; however, the non-overlapping area unveiled several differences in their diets or foraging strategies, conveying that these two pandas have undergone trophic niche differentiation during their long-term coexistence process. Herbivorous mammals generally demonstrate a negative correlation between body size and the quality of diet, where large herbivores consume foods that are nutritionally deficient, while small ones consume foods that are more nutritionally rich [61]. Giant pandas, having larger body size, used sites with lower densities of shrubs, fallen logs, and bamboo culms, as feeding and moving in this more open microhabitat could reduce energy expenditures. Saving energy is particularly important for the giant panda because its daily energy intake only marginally exceeds expenditures [62,63]. In contrast, smaller red pandas usually walk on the fallen logs, branches of shrubs, and tree stumps, which give them easy access to bamboo leaves [63,64,65]. In the case where, along with body size, the increase in encounter probability is less favorable and the handling time is more favorable than metabolic costs, then body size constitutes a trade-off between search efficiency (which favors a smaller body size) and handling efficiency (which favors a larger one) [66]. The activity level of giant pandas is often lower than that of red pandas, and red pandas are able to move around flexibly by using branches and other tools to feed on higher bamboo leaves; giant pandas rely on consuming a large amount of food to survive, while red pandas are relatively more selective for their diet [67,68,69]. Giant pandas utilize almost every part of bamboo species, including leaves, shoots, culms and branches, whereas red pandas only use bamboo leaves and shoots. Despite the fact that both species employed shoots and leaves, there was a significant difference in their utilization pattern; bamboo leaves constituted 89.9% of the red panda’s annual diet, while it was only 34.7% for the giant panda. Also, the giant panda had a preference for taller shoots, in contrast to the red panda [70]. Furthermore, these two pandas adopt different foraging strategies to meet their nutritional requirements; giant pandas adhere to bamboo foraging, and in the season of bamboo sprouting, they increase the proportion of shoot intake [71], while red pandas incorporate other food items in the summer and fall, such as berries, mosses, mushrooms, flowers and so on.

The niche width and the overlapping degree of the two species are not only affected by food resources, but also related to environmental resources. This means that the differentiation of trophic niche between giant and red pandas is, to some extent, related to their separation in microhabitats. The selection of specific microhabitats by each animal reflects ecological adaptation dependent on behavior linked to its diet, body size, energy metabolism, and other factors, ensuring successful survival and reproduction by minimizing competition. In the past, many researchers have speculated on the trophic niche partition between giant pandas and red pandas through field observations, infrared camera surveys, and other approaches focused on microhabitat factors. Giant pandas preferred to move and forage on slopes with gentler gradients [13,72], while the flexible red pandas selected steeper slopes at lower elevations and areas with higher amounts of sunlight [16,19], allowing them to better avoid predators [73,74]. The habitat selection of red pandas in upland areas with less human activity can also provide the benefit of better detecting danger during the cub rearing season [75]. These characteristics of habitat differentiation are consistent with the results of stable isotope trophic niche and width analysis. Different patterns of resource utilization and microhabitat selection between the giant and red panda minimize the competition, which is crucial to the co-existence of both species.

## 5. Conclusions

In this study, using the stable isotope technique, we found that (1) there were significant differences in the nitrogen stable isotope ratios of the hair keratin between *A. styani* and *A. fulgens*, owing to the staple food growing in their different altitude ranges. (2) The trophic niche widths of three genetic populations in *A. styani* were calculated and compared, and combined with the fecal analysis, the results suggested that different populations had diverse abundance of available food resources and experienced varying levels of competition stress. (3) Integrated with the stable isotope compositions of other animals distributed in the same area, the relative trophic position of SC red pandas was confirmed to be a lower level than the carnivores and herbivores in the ecosystem. (4) Lastly, the isotopic trophic niche width of SC red pandas was two times larger than that of SC giant pandas and there is considerable overlap as well as separated areas between them, which was closely related to the foraging strategies and microhabitat selection of the giant and red panda. We believe that our conclusions could provide some benefit for the conservation of red pandas, as they provide a new insight and scientific basis for management and policy development from stable isotopes. Consequently, instead of simply taking the mountain or geographic area as the unit of rough management, the trophic niche width and genetic information should be considered for dividing the management units in the future.

## Figures and Tables

**Table 1:** Carbon and nitrogen stable isotopic composition in hair keratin of red pandas.

Species	Group	N	δ^13^C (‰)	δ^13^C (‰) Range	δ^15^N (‰)	δ^15^N (‰) Range
*Ailurus styani*	EH-GLG	53	−25.19 ± 0.95	−27.25~−23.11	1.19 ± 1.13	−0.97~3.45
	XXL-LS	10	−23.53 ± 1.15	−24.50~−20.53	1.43 ± 1.28	−0.97~3.31
	QL	8	−24.70 ± 0.57	−25.34~−23.82	1.95 ± 1.13	−0.62~2.78
	Unclassified	15	−24.43 ± 1.20		−0.10 ± 1.17	
	Total	86	−24.73 ± 1.26	−27.25~−20.41	1.30 ± 1.09	−0.97~3.45
*Ailurus fulgens*	C-W Nepal	5	−24.22 ± 1.40	−26.02~−22.13	0.18 ± 0.49	−0.49~0.73
	E-Nepal	5	−24.61 ± 0.87	−25.39~−23.20	−1.30 ± 0.77	−1.99~0.05
	Unclassified	6	−24.31 ± 1.65		0.87 ± 0.86	
	Total	16	−24.43 ± 1.20	−26.02~−22.13	−0.10 ± 1.17	−1.99~0.73

## Data Availability

All data can be found in the article.

## Supplementary Materials

The following supporting information can be downloaded at: https://www.mdpi.com/article/10.3390/ani14233512/s1, Figure S1: Composition of red panda feces in Liziping National Nature Reserve across different seasons; Figure S2: Carbon and nitrogen stable isotope ratios and trophic niche ellipses of red pandas (red) and giant pandas (black) from Sichuan province.
