# Supplementary figures and images for: Comparison Study on the Trophic Niche of Red Pandas Using Stable Isotope Analysis
Source: Animals (Basel). 2024 Dec 5;14(23):3512. doi: 10.3390/ani14233512 (PMC11639846; doi:10.3390/ani14233512)

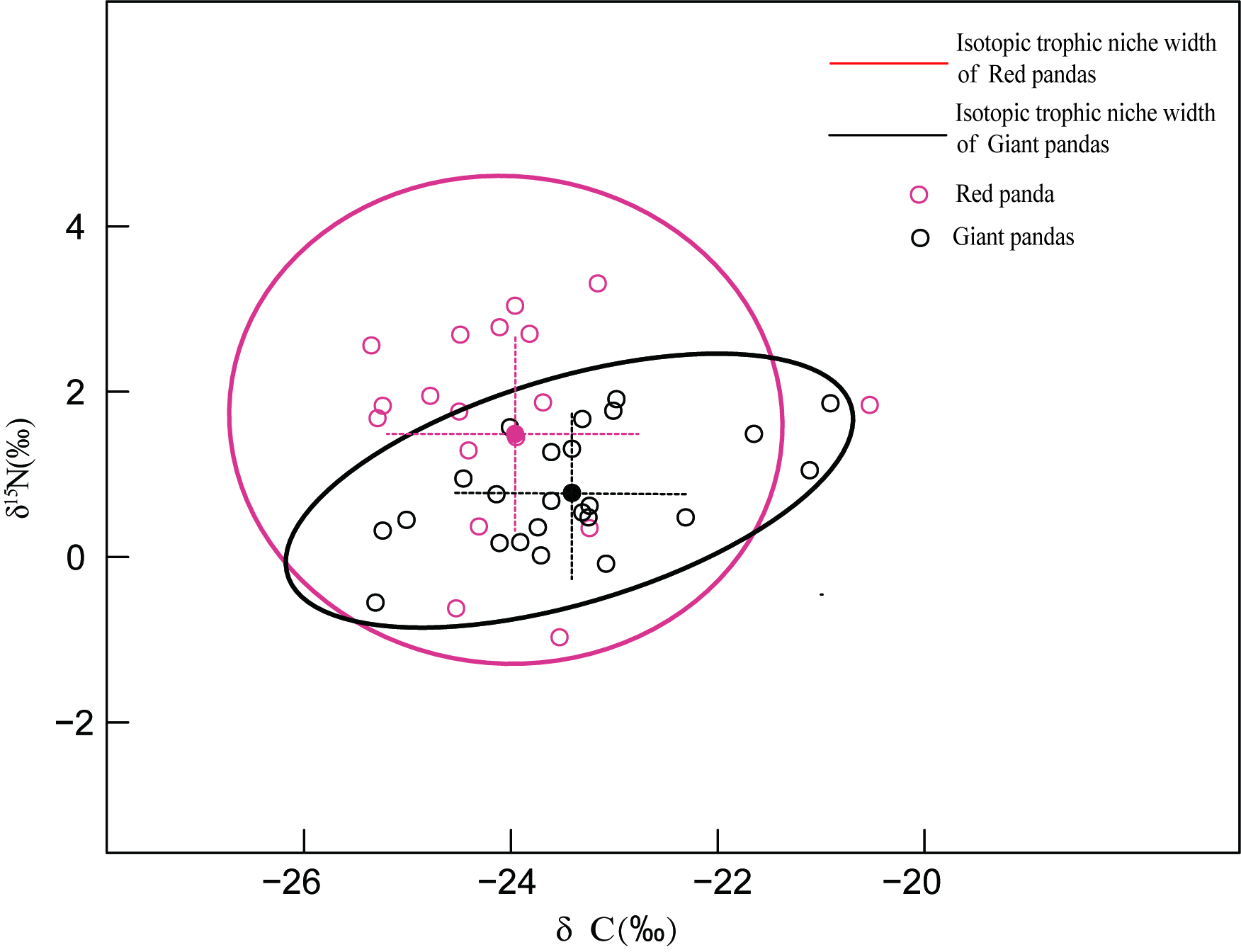

Supplement: Supplementary file 1 [file animals-14-03512-s001.zip › Figure S1.tif]

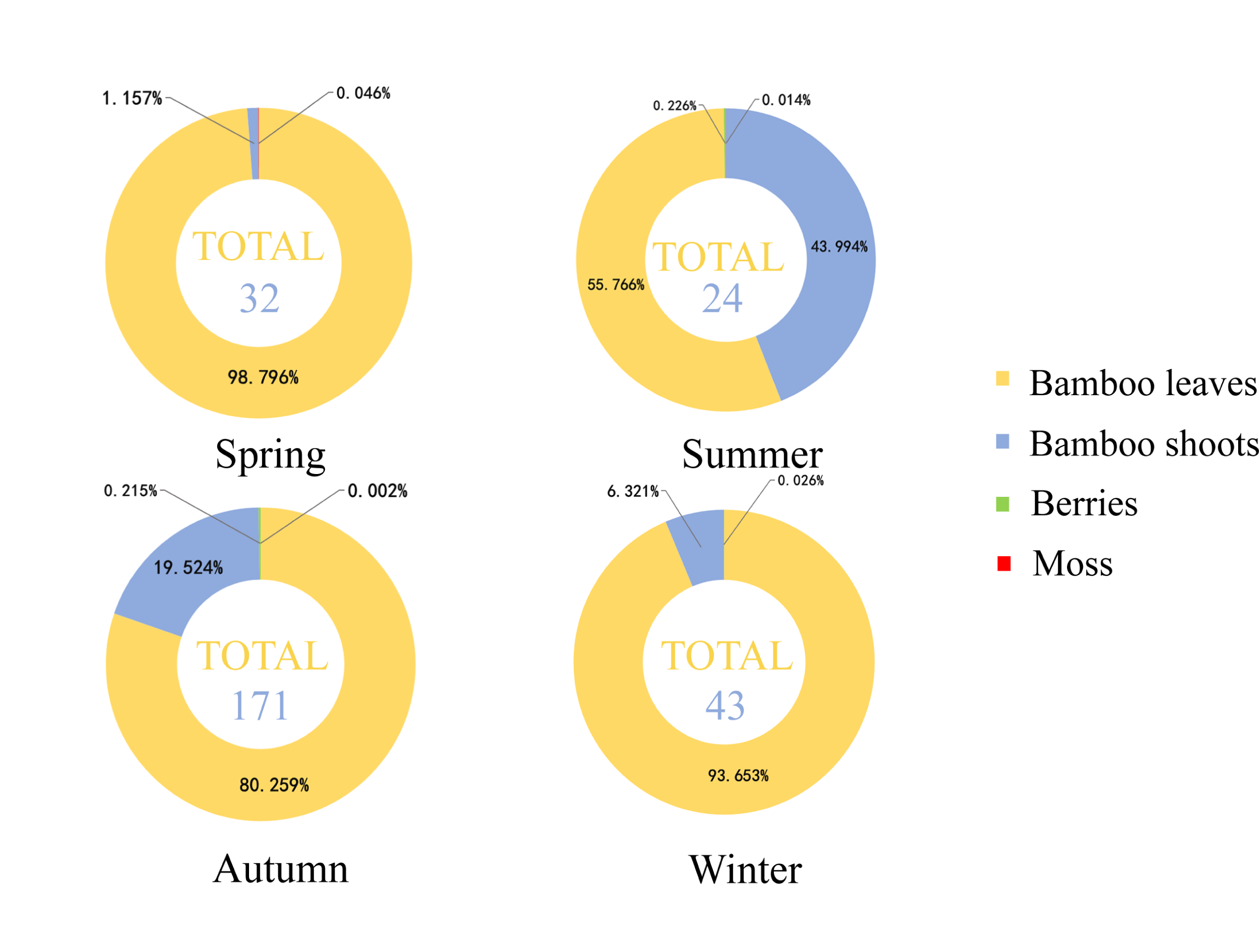

Supplement: Supplementary file 1 [file animals-14-03512-s001.zip › Figure S2.tif]
